# Editorial: Cardiovascular Remodeling in Aging and Disease

**DOI:** 10.3389/fphys.2022.867185

**Published:** 2022-03-07

**Authors:** Jochen Steppan, Daniel Nyhan, Lakshmi Santhanam

**Affiliations:** ^1^Department of Anesthesiology and Critical Care Medicine, Johns Hopkins University, Baltimore, MD, United States; ^2^Department of Biomedical Engineering, Johns Hopkins University, Baltimore, MD, United States; ^3^Department of Chemical and Biomolecular Engineering, Johns Hopkins University, Baltimore, MD, United States

**Keywords:** vasculature, remodeling, stiffness, aging, cardiac, hypertension, pulmonary

Cardiovascular remodeling is a key feature of arterial aging and cardiovascular disease and a direct contributor to a shortened lifespan (van Popele et al., 2001; Vlachopoulos et al., 2010; AlGhatrif et al., 2013; Niiranen et al., 2016). Recent advances have shown that vascular stiffening affects all vascular beds, encompasses functional perturbations in resident cells, produces structural/compositional changes in the matrix, and is directly linked to cardiac remodeling and impaired ventricular-arterial coupling (Nichols et al., 1985; Qiu et al., 2010; Santhanam et al., 2010; Lacolley et al., 2018; Steppan et al., 2019; Wang et al., 2021). These changes can occur over many years, as in natural aging, or can be accelerated by factors such as hypertension, diabetes, smoking, and hyperlipidemia (Niiranen et al., 2016; Boutouyrie et al., 2021; Mitchell, 2021). In the pulmonary circulation, vascular remodeling that develops with pulmonary arterial hypertension is now an area of active investigation. In the absence of clear targetable mechanisms, diet and exercise continue to be the mainstay of therapy (Steppan et al., 2012, 2014). This Frontier's Research Topic has collated nine contributions (three reviews and six original research articles) spanning basic science and clinical research in both the systemic and pulmonary circulation.

In a comprehensive review on vascular stiffness, Vatner et al. discuss regional changes in stiffness along the aorta, the role of sex, the transferability of animal studies to the clinical setting, and global population variations. They describe extracellular matrix derangement and endothelial cell (EC) and smooth muscle cell (SMC) dysfunction at the cellular and molecular levels. In the context of molecular mechanisms, researchers increasingly recognize the contributions of the “vascular triad,” comprising reactive oxygen species, inflammation, and vascular dysfunction, to the development of sustained hypertension. Ranadive et al. review these known elements and extend the discussion to include inducible nitric oxide synthase, hydrogen peroxide, hydrogen sulfide, NF-κB, and nuclear factor activated T cells as a set of “unusual suspects” potentially contributing to the pathogenesis of hypertension and the associated vascular dysfunction. The final review focuses on micro-RNAs (miR) as intriguing and important regulators of arterial stiffening. Baraban et al. review the role of miRs in stiffening of the large arteries and their potential for therapeutic intervention (Hori et al., 2017). They highlight prospective areas of research to determine regulation of miRs and their exact mechanism of action in the aging vessel.

In the systemic circulation, hyperlipidemia and atherosclerosis accelerate arterial aging. Endothelial dysfunction induced by oxidized low-density lipoprotein plays a critical role in the pathobiology of atherosclerosis and the associated vascular remodeling. A chief element of this is gene expression changes in ECs linked to histone acetylation, which is regulated by histone acetylases and histone deacetylases (HDACs). Nomura et al. demonstrate that HDAC6 inhibition with tubacin attenuates atherosclerosis in a mouse model. They further show that NEDDylation is a potential mechanism by which HDAC6 function is regulated in the atheromatous environment.

While rodent models have clear advantages over human studies, such as feasibility of mechanistic studies and lower cost, the differences in vascular aging across species must be considered when translating the results (Vatner et al.). In novel work using fluid-structure interaction models of the human and mouse aorta, Hopper et al. demonstrate differences in the indices of vascular stiffness in the young and distinct regional differences in the aged aorta between mice and humans. Thus, results from mouse studies must be interpreted within the context of these differences. Another challenge to research in mice is that their small size and fast heart rate make echocardiographic studies particularly challenging (Rottman et al., 2007). In a human echocardiography study, Heidemann et al.  evaluate the relationship between atrial septal mobility, which is related to embolic stroke, and aortic root dilation (a disease of the vessel wall). They show that increases in atrial septal mobility correlate with increases in aortic root diameter, suggesting that aortic root diameter should be evaluated in patients with increased atrial septal mobility.

Vascular stiffness measurement is also important in patients with aortic aneurysms, in particular, after endovascular repair. Using a clinical cohort of patients undergoing endovascular aortic repair, Hori et al. compared five different endovascular devices. They show, for the first time in a clinical population, that the different devices do affect aortic pulse wave velocity (PWV), the gold-standard index of *in vivo* arterial stiffness. Notably, treatment length was more important than treatment site.

Finally, pulmonary arterial remodeling is increasingly recognized as important to pulmonary hypertension and right heart failure. In an elegant study, Manning et al. evaluated the underlying role of vascular remodeling in pulmonary hypertension by using single cell RNA sequencing and multiphoton microscopy to determine microstructural features, associated changes in material stiffness, and vasoactive capacity. They show that pulmonary arteries of hypoxic mice exhibit transcriptome changes, SMC phenotype alterations, and endothelial proliferation, likely representing endothelial-to-mesenchymal transition. Interestingly, they suggest that the increased material stiffness noted in the pulmonary artery is due to collagen reorientation rather than fibrosis. Furthermore, pulmonary PWV increased. Thus, they uncovered an important positive feed-forward loop driving pulmonary hypertension. In a separate study investigating the mechanisms underlying vascular remodeling in pulmonary hypertension, Yun et al. evaluated factors modulating pulmonary arterial SMC and EC hyperplasia and migration. Using the Sugen5416/hypoxia rat model of pulmonary hypertension that recapitulates the main features of human pulmonary arterial hypertension, they show that aquaporin (AQP1) protein levels are increased in both SMCs and ECs. Using loss- and gain-of-function approaches, they establish a key role for increased AQP1 expression in the enhanced migration and proliferation of pulmonary artery SMCs and pulmonary artery ECs, both prominent features of severe pulmonary arterial hypertension.

We hope the reader finds this Research Topic to be a useful reference in understanding cardiovascular remodeling in aging and disease, in particular the underlying mechanisms, the different diseases that cause or accelerate mechanical decline of the cardiovascular system, and pitfalls in data analysis and interpretation.

## Author Contributions

All authors listed have made a substantial, direct, and intellectual contribution to the work and approved it for publication.

## Funding

This work was supported by a NHLBI grant R01HL148112 (LS) and a NHLBI grant K08HL145132 (JS).

## Conflict of Interest

The authors declare that the research was conducted in the absence of any commercial or financial relationships that could be construed as a potential conflict of interest.

## Publisher's Note

All claims expressed in this article are solely those of the authors and do not necessarily represent those of their affiliated organizations, or those of the publisher, the editors and the reviewers. Any product that may be evaluated in this article, or claim that may be made by its manufacturer, is not guaranteed or endorsed by the publisher.

## References

[B1] AlGhatrif M. Strait J. B. Morrell C. H. Canepa M. Wright J. Elango P.. (2013). Longitudinal trajectories of arterial stiffness and the role of blood pressure: the Baltimore Longitudinal Study of Aging. Hypertension 62, 934–941. 10.1161/HYPERTENSIONAHA.113.01445 24001897 PMC3880832

[B2] Boutouyrie P. Chowienczyk P. Humphrey J. D. Mitchell G. F. (2021). Arterial stiffness and cardiovascular risk in hypertension. Circ. Res. 128, 864–886. 10.1161/CIRCRESAHA.121.318061 33793325

[B3] Hori D. Dunkerly-Eyring B. Nomura Y. Biswas D. Steppan J. Henao-Mejia J.. (2017). miR-181b regulates vascular stiffness age dependently in part by regulating TGF-beta signaling. PLoS ONE 12, e0174108. 10.1371/journal.pone.0174108 28323879 PMC5360327

[B4] Lacolley P. Regnault V. Avolio A. P. (2018). Smooth muscle cell and arterial aging: basic and clinical aspects. Cardiovasc. Res. 114, 513–528. 10.1093/cvr/cvy009 29514201

[B5] Mitchell G. F. (2021). Arterial stiffness in aging: does it have a place in clinical practice?: recent advances in hypertension. Hypertension 77, 768–780. 10.1161/HYPERTENSIONAHA.120.14515 33517682

[B6] Nichols W. W. O'Rourke M. F. Avolio A. P. Yaginuma T. Murgo J. P. Pepine C. J.. (1985). Effects of age on ventricular-vascular coupling. Am. J. Cardiol. 55, 1179–1184. 10.1016/0002-9149(85)90659-9 3984897

[B7] Niiranen T. J. Kalesan B. Hamburg N. M. Benjamin E. J. Mitchell G. F. Vasan R. S. (2016). Relative contributions of arterial stiffness and hypertension to cardiovascular disease: the framingham heart study. J. Am. Heart Assoc. 5, e004271. 10.1161/JAHA.116.004271 27912210 PMC5210358

[B8] Qiu H. Zhu Y. Sun Z. Trzeciakowski J. P. Gansner M. Depre C.. (2010). Short communication: vascular smooth muscle cell stiffness as a mechanism for increased aortic stiffness with aging. Circ. Res. 107, 615–619. 10.1161/CIRCRESAHA.110.221846 20634486 PMC2936100

[B9] Rottman J. N. Ni G. Brown M. (2007). Echocardiographic evaluation of ventricular function in mice. Echocardiography 24, 83–89. 10.1111/j.1540-8175.2006.00356.x 17214630

[B10] Santhanam L. Tuday E. C. Webb A. K. Dowzicky P. Kim J. H. Oh Y. J.. (2010). Decreased S-nitrosylation of tissue transglutaminase contributes to age-related increases in vascular stiffness. Circ. Res. 107, 117–125. 10.1161/CIRCRESAHA.109.215228 20489165

[B11] Steppan J. Sikka G. Jandu S. Barodka V. Halushka M. K. Flavahan N. A.. (2014). Exercise, vascular stiffness, and tissue transglutaminase. J. Am. Heart Assoc. 3, e000599. 10.1161/JAHA.113.000599 24721796 PMC4187484

[B12] Steppan J. Tran H. Benjo A. M. Pellakuru L. Barodka V. Ryoo S.. (2012). Alagebrium in combination with exercise ameliorates age-associated ventricular and vascular stiffness. Exp. Gerontol. 47, 565–572. 10.1016/j.exger.2012.04.006 22569357 PMC4359688

[B13] Steppan J. Wang H. Bergman Y. Rauer M. J. Tan S. Jandu S.. (2019). Lysyl oxidase-like 2 depletion is protective in age-associated vascular stiffening. Am. J. Physiol. Heart Circ. Physiol. 317, H49–H59. 10.1152/ajpheart.00670.2018 31002285 PMC6692735

[B14] van Popele N. M. Grobbee D. E. Bots M. L. Asmar R. Topouchian J. Reneman R. S.. (2001). Association between arterial stiffness and atherosclerosis: the Rotterdam Study. Stroke 32, 454–460. 10.1161/01.STR.32.2.454 11157182

[B15] Vlachopoulos C. Aznaouridis K. Stefanadis C. (2010). Prediction of cardiovascular events and all-cause mortality with arterial stiffness: a systematic review and meta-analysis. J. Am. Coll. Cardiol. 55, 1318–1327. 10.1016/j.jacc.2009.10.061 20338492

[B16] Wang H. Chen J. Jandu S. Melucci S. Savage W. Nandakumar K.. (2021). Probing tissue transglutaminase mediated vascular smooth muscle cell aging using a novel transamidation-deficient Tgm2-C277S mouse model. Cell Death Discov. 7, 197. 10.1038/s41420-021-00543-8 34326316 PMC8322091

